# Family caregiver related factors contributing to role strain among adult patients with cancer at a National Referral Hospital in Kenya: cross-sectional study

**DOI:** 10.11604/pamj.2024.49.86.32659

**Published:** 2024-11-22

**Authors:** Morris Murithi Muriuki, Sherry Oluchina, Bernard Wambua Mbithi

**Affiliations:** 1 School of Nursing, Jomo Kenyatta University of Agriculture and Technology, Nairobi, Kenya

**Keywords:** Family caregiving, role strain, caregiving strain, caregiver strain, cancer caregiving, cancer patients

## Abstract

**Introduction:**

cancer care involves a long-term treatment plan with a demand for continuous care which currently is largely being offered as an outpatient service and worldwide family caregiving is becoming the backbone for long-term care delivery. There is a paucity of information regarding family caregivers of patients with cancer in Africa. Therefore, this study aimed to determine family caregiver-related factors contributing to role strain among family caregivers of adult patients with cancer, this being a first step in designing the best strategies to mitigate the role strain.

**Methods:**

the study adopted an analytical cross-sectional design involving 255 systematically sampled family caregivers of adult patients attending Kenyatta National Hospital outpatient cancer treatment clinic between February and March 2020. Both quantitative and qualitative data were collected. Quantitative data from a structured questionnaire and Modified Caregiver Strain Index (MCSI) tool was analyzed by deriving descriptive statistics and ordinal logistic regression was performed to derive the relationship between the independent variables and the dependent variable. Quantitative data was presented by use of tables and charts. SPSS software version 25 was utilized in data analysis. Qualitative data from focused group discussion interviews was analyzed thematically.

**Results:**

from the study findings, the family caregiver-related factors associated with role strain include being married (AOR=0.49, 95% CI 0.252-0.960, p=0.038); unemployed (AOR=3.29, 95% CI 1.833-5.894, p=0.001); providing care for <5 hours (AOR 0.40, 95% CI 0.227-0.715, p=0.002); lack of perception of strain related to social isolation (AOR=0.20, 95% CI 0.075-0.541, p=0.001), disturbed sleep cycle (AOR=0.30, 95% CI 0.141-0.641, p=0.002), social support (AOR=0.13, 95% CI 0.051-0.391, p=0.001) and costly transport costs (AOR=0.32, 95% CI 0.121-0.816, p=0.017).

**Conclusion:**

there are various family caregiver-related factors associated with the role strain. This therefore calls for healthcare practitioners to pro-actively consider FCGs for psychological counseling, social support groups, health education, and provision of literature materials on self-care and self-financial empowerment, spiritual support and referral for financial support if available.

## Introduction

Cancer is a global health burden exerting physical, psychological, and financial strain [1] on every individual, family, and healthcare system. Currently, cancer care and treatment are largely being offered as an outpatient service than an inpatient service [2] and this has partly shifted the burden of care from healthcare professionals to patients and their family caregivers (FCGs) [3] setting the stage for home-based family caregiving. There are various factors contributing to caregiving strain which include socio-demographic characteristics, and psychological and financial factors [4]. Family caregivers are also affected psychologically, socially, physiologically and financially during caregiving [5]. Indeed, family caregivers (FCGs) get psychologically upset but keep concealing their emotional devastation from their patients and other family relatives. This is made worse by disrupted social life, family-role conflict, disturbed sleep cycle, and financial strain [6]. Despite the complex roles that family caregivers play, healthcare professionals focus only on addressing patient´s needs, and the family caregiver´s needs are never addressed [7]. Further, many cancers treatment centers do not provide comprehensive programs to support family caregivers in caregiving [8] and they also lack a formalized mechanism for integrating the FCGs in the unit of care.

There is a research gap regarding FCGs and not much is known about the caregiving challenges faced by the FCGs in Africa [9]. This implies the need to determine the factors contributing to role strain or the caregiving strain, this being a first step in designing effective interventional programs to mitigate the role strain experienced by the family caregivers. Therefore, this study aimed to determine the family caregiver-related factors contributing to role strain among family caregivers of adult patients with cancer.

## Methods

Study design: this study utilized analytical cross-sectional study comprising both quantitative and qualitative approaches in data collection.

Study setting: this study was conducted at Kenyatta National Hospital (KNH) which is the largest national teaching and referral hospital in Kenya as well as in the eastern and central Africa region. It is located within Nairobi County. Kenyatta National Hospital receives referrals from across the whole country and is the largest government hospital that offers comprehensive cancer treatment and care services which comprise the main cancer treatment modalities (surgery, chemotherapy, radiotherapy) and follow-up care. Kenyatta National Hospital was an ideal site for this study since it receives patients with cancer and their family caregivers from across the whole country who have to cover long geographical distances among other strains in order to access the comprehensive cancer treatment and care services that are only available in very few healthcare facilities in the country. The study was conducted at the KNH cancer treatment outpatient clinic between February and March 2020. The clinic attends approximately to 1000 patients who are booked every week for routine treatment and follow-up care (cancer treatment center health information department statistics, 2018).

Study population and sample size: the study population comprised of family caregivers to patients attending Kenyatta national hospital cancer outpatient treatment clinic. The annual data for the year 2018/2019 indicated that 9098 patients received chemotherapy or a combination of chemotherapy and radiotherapy (cancer treatment centre health information department statistics, 2019). This indicated that on average 758 patients were administered chemotherapy or chemo-radiation every month and since almost every patient is accompanied by a family caregiver, the estimated study population corresponded to approximately 758 family caregivers. A sample size of 255 family caregivers were recruited utilizing a systematic sampling method with the patient booking register forming the sampling frame. Sample size calculation was based on Cochran's sample size formula [10]:


$$n=\frac{Z^{2}dq}{d^{2}}$$

Since the target population was less than 10,000, the sample size was adjusted using the finite population correlation factor formula:


$$nf=\frac{n}{1+n/N}$$

Inclusion criteria was based on: men and women who were over 18 years, caring for a patient on chemotherapy or a combination with radiotherapy (chemo-radiation) and if more than one family caregiver; closest relative who resides near the patient and who has provided care the longest to the patient at least two weeks and above. Exclusion criteria was based on: Family caregivers that were being paid for care rendered, those who were very sick or mentally incapable, and family caregivers who were health care professionals.

Study variables: dependent variable or the outcome variable for this study was the level of role strain which was categorized as mild, moderate, and severe role strain. Independent variables included family caregiver socio-demographic and caregiving characteristics (sex, age, education level, marital status, employment status, monthly income, co-residency with the patient, relationship with the patient, duration of caregiving, hours of care provision, and membership to a social support group); psycho-social related factors (perceived strain-related to social isolation, disturbed sleep cycle, lack of social support and psychological distress) and financial related factors (perceived strain related to transport costs, accommodation costs, drug costs, investigation costs and general financial strain).

Data collection and data tools: both quantitative and qualitative data were collected. Researcher administered a structured questionnaire and the Modified Caregiver Strain Index (MCSI) tool [11] was used in collecting quantitative data. Modified caregiver strain index tool is composed of 13 questions aimed at measuring strain due to care provision and is a validated tool with an internal reliability coefficient of 0.90 and a test-retest reliability coefficient of 0.88 [11]. The MCSI tool is scored by awarding 2 points for each “yes” and 1 point for each “sometimes” response. The sum score of the items ranges from 0 to 26. High scores reflect a high level of strain. The level of role strain was categorized as mild strain (0-8), moderate strain (9-17) and severe strain (18-26). Similar categorization of strain has been adopted by another study [12] which was conducted in India.

The structured questionnaire collected data on socio-demographic and caregiving-related characteristics and psycho-social and financial-related factors. Psycho-social and financial factors; were each assessed using a 5-item likert scale where the respondents were requested to rate each item statement on a 5-point scale from strongly disagree=1, disagree=2, somehow agree=3, agree=4, and strongly agree=5. However, the first two scores (1 and 2) were an indicator that the respondents were totally not in support of the statement or did not perceive any strain. The scores of 3, 4, and 5 were taken as indicators that the respondents perceived the strain, though at different levels. For data analysis purposes the scores of 1 and 2 were computed together under disagree which meant that the respondents did not perceive any strain while a score of 3 meant that the respondents slightly perceived the strain. Scores of 4 and 5 were computed together under agree which meant that the respondents totally perceived the strain. The structured questionnaire was also pretested prior to the actual commencement of the study. This involved a sample of 10% of the study sample size among family caregivers of adult patients admitted at Kenyatta National Hospital Oncology Ward. This data was also utilized in calculating the reliability coefficients for the Likert scales in the structured questionnaire where a 5 item likerts scale on psycho-social factors had a Cronbach alpha of 0.75. Similarly, that of a 5-item scale on financial factors was alpha=0.75. Focus group discussion guide was used in collecting qualitative data from three focus group discussions each consisting of a minimum of eight respondents who were purposively sampled.

Data analysis and presentation: quantitative data from structured questionnaire and Modified Caregiver Strain Index (MCSI) tool was analyzed by deriving descriptive statistics and ordinal logistic regression was performed to derive the relationship between the independent variables and the dependent variable (level of role strain; mild, moderate and severe role strain). To derive the relationship between the dependent variable (level of role strain; mild, moderate, and severe role strain) and independent variables (predictor variables); ordinal logistic regression was performed with confidence interval set at 95% (p-value < 0.05 was considered significant). To control for confounders all the predictor variables that were significant after bivariate ordinal logistic regression were fitted in a multivariate ordinal logistic regression. Quantitative data was presented by use of tables and charts. SPSS software version 25 was utilized in data analysis. Qualitative data from focused group discussion interviews was analyzed thematically.

Ethical considerations: this study was approved by Kenyatta National Hospital and the University of Nairobi Ethics and Review Committee (KNH/UON ERC). Clearance to conduct the study was also obtained from the head of the department Cancer Treatment Centre (CTC) at Kenyatta National Hospital. A research permit was also obtained from the National Commission for Science, Technology, and Innovation (NACOSTI). Informed consent was sought from study respondents before data collection. Privacy and confidentiality were also maintained throughout during data collection and analysis.

## Results

**Figure 1:**
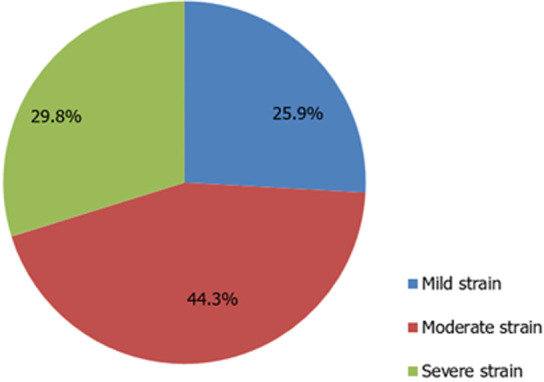
level of strain among family caregivers

**Level of family caregiver role strain:** a total of 255 family caregivers were recruited in this study representing a 100% response rate. Figure 1 illustrates the level of role strain based on assessment using the MCSI. The results revealed that mild strain (n=66; 25.9% (95% CI: 20.6 to 31.7)) and severe strain (n=76; 29.8% (95% CI: 24.3 to 35.8)) were experienced in almost equal proportions while over two-fifth (n=113; 44.3% (95%CI: 38.1 to 50.6)) of the respondents experienced moderate strain.

**Socio-demographic and caregiving related characteristics of the family caregivers:** Table 1 shows the socio-demographic characteristics of the family caregivers and caregiving-related characteristics that were assessed during the study. Majority (57.6%, n=147) were females, most (43.1%, n=110) had attained a primary level of education, and over half (57.3%, n=146) of the respondents were unemployed. On caregiving-related characteristics; the majority (91.0%, n=232) of the respondents resided with the patient within the same homestead, most (36.9%, n=94) cared for a spouse and majority (74.5%, n=190) provided care for a period of less 5 hours per day.

**Table 1:** family caregiver socio-demographic and caregiving-related characteristics (n=255)

Variable	Category	N (%)
**Family caregiver socio-demographic characteristics**		
Sex	Male	108 (42.4%)
	Female	147(57.6%)
Age	18-35 years (young adult)	31(12.2%)
	36-60 years (middle-aged)	212(83.1%)
	Over 61 years (elderly)	12(4.7%)
Education	Primary	110(43.1%)
	Secondary	106(41.6%)
	Tertiary/college	39(15.3%)
Marital status	Married	206(80.8%)
	Not married (single, separated, widowed)	49(19.2%)
Employment status	Unemployed	146(57.3%)
	Employed (full-time, part-time, self-employed)	109(42.8%)
Monthly income	Low income (<20000Ksh)	219(85.9%)
	Moderate income (>20,000-50,000Ksh)	32(12.5%)
	High income (>50,000Ksh)	4(1.6%)
**Caregiving related characteristics**		
The caregiver resides with the patient	No	23(9%)
	Yes	232(91.0%)
Relationship with patient	Parent/in-law parent	87(34.1%)
	Spouse/partner	94(36.9%)
	Son/daughter	10(3.9%)
	Friend/neighbor	9(3.5%)
	Brother/sister	55(21.6%)
Duration of caregiving to patient	>2 week-<1 year	171(67.1%)
	>1 year-3 years	80(31.4%)
	>3 years	4(1.6%)
Hours of care provision per day	<5 hours	190(74.5%)
	>5 hours	65(25.5%)
Membership in a social support group	Yes	21(8.2%)
	No	234(91.8%)

**Association between socio-demographic/caregiving related characteristics and family caregiver role strain**: Table 2 shows socio-demographic and caregiving-related characteristics associated with family caregiver role strain after multivariate ordinal logistic regression was performed. The results revealed that family caregivers who were married were 51% less likely to experience severe role strain as compared to those who were not married (OR=0.49, 95% CI 0.252-0.960, p=0.038). Likewise, those who were unemployed were over three times more likely to experience a severe role strain compared to their counterparts (OR=3.29, 95% CI 1.833-5.894, p=0.001). This study also established that family caregivers providing care for less than 5 hours per day were 60% less likely to experience severe role strain than those who provided care for more than 5 hours per day (OR 0.40, 95% CI 0.227-0.715, p=0.002). This study did not establish any significant relationship between the family caregiver´s age, level of education, monthly income, co-residency with the patient, and the relationship with the patient with the role strain experienced by family caregivers of adult patients with cancer.

**Table 2:** results of ordinal logistic regression analysis on association between socio-demographic/caregiving characteristics and role strain experienced by family caregivers of adult patients with cancer

Variable	Beta Estimate	Sig.	Crude O.R		Sig.	Adjusted O.R	95% CI	
							Lower	Upper
Bivariate ordinal logistic regression			Beta estimate	Multivariate ordinal logistic regression				
**Sex**								
Male (female; Ref)	0.032	0.893	1.03					
**Age**								
18-35 years (young adult)	-1.729	**0.010**	0.18	-0.453	0.536	0.64	0.151	2.670
36-60 years (middle-aged)				-0.188	0.765	0.83	0.242	2.840
Over 61 years (elderly) (Ref)	-1.218	**0.038**	0.30					
**Education level**								
Primary	0.970	**0.006**	2.60	0.573	0.145	1.80	0.821	3.827
Secondary	0.111	0.751	1.12	-0.123	0.749	0.90	0.416	1.879
Tertiary (Ref)								
**Marital status**								
Not married		**0.010**	0.46	-0.710	**0.038**	0.49	0.252	0.960
Married (single, separated, widowed) (Ref))								
**Employment status**	-0.775							
Unemployed				1.190			1.833	5.894
Employed (full-time, part-time, self-employed) (Ref)					**0.001**	3.29		
**Monthly income level**	1.415	**0.001**	4.12					
Low income (<20,000Ksh)	1.345	**0.024**	3.83	0.112	0.869	1.12	0.295	4.246
Moderate income (>20,000-50,000Ksh)		0.749		0.116	0.877	1.12	0.257	4.903
High Income (>50,000Ksh) (Ref)	0.222							
**FCG co-residency with the patient**			1.25					
No (Yes; Ref)	-0.845					0.60		1.659
**Family caregiver relationship with the patient (who the patient is to the FCG)**		**0.041**	0.43	-0.510	0.325		0.217	
Parent/parent-in-law	0.356	0.270	1.43	-0.183	0.598	0.83	0.421	1.647
Spouse/partner	0.880	**0.006**	2.41	0.087	0.818	1.10	0.520	2.289
Son/daughter	-0.283	0.661	0.80	-0.942	0.167	0.40	0.102	1.484
Friend/neighbor Brother sister (Ref)	-0.658			-0.455	0.587	0.63	0.123	3.267
**Duration of caregiving**		0.338	0.52					
<1 year	-0.806	0.398	0.50					
>1 year to 3 years	-0.247							
>3 years (Ref)								
**Hours of caregiving per day**		0.798	0.80					
< 5 hours (>5 hours; Ref)	-1.021						0.227	
**Membership to a social support group for FCG of adult patients with cancer**		**0.001**	0.40	-0.909	**0.002**	0.40		0.715
Yes	-0.487	0.252	0.61					
No (Ref)								


Notes: only significant variables (p=>0.05) after bivariate logistic regression were considered for multivariate logistic regression; the dependent variable was the level of role strain (1= mild strain, 2= moderate strain, 3= severe strain (Ref)

### Family caregiver-related psycho-social and financial factors contributing to role strain experienced by the family caregivers of adult patients with cancer

Table 3 shows family caregiver psycho-social and financial factors assessed during the study. The results have also been presented alongside thematic analysis of the qualitative data. The results revealed that the majority (80.8%, n=206) of FCGs totally perceived (those who agreed) social isolation as a strain. Likewise, during focus group discussions, the majority of respondents indicated that there was no time for social activities as most of their time was utilized taking care of the patient. Others also felt socially isolated/stigmatized by their social groups and friends as supported by the following quotes from some of the participants: “Cancer treatment consumes a lot of time such that I have little time to attend my social group meetings” (family caregiver (FCG) No.2, FGD.1). “We can not have a social life with other people, majority of people isolate us” (FCG No.11, FGD.2). The results also revealed that the majority (74.1%, n=189) of FCGs totally perceived lack of social support as a strain. Indeed, during focus group discussions majority of FCGs indicated that they experienced lack of social support from their families including friends and neighbors. Others experienced job challenges or job loss related to unsupportive employers as supported by the following quotes from some of the participants: “My neighbors used to assist in taking care of my children when I bring the patient to the clinic, but of late they feel it is a big bother, even neighbors have gotten tired with me” (FCG No.5, FGD.1). “Caring for my sick sister has brought some misunderstanding between me and my husband; he feels that I have brought a big burden to his family” (FCG No.21, FGD.3). “I have to accompany my patient every time she attends the clinic, on her own she can not make it, the employer has told me to choose between the job and my sick wife” (FCG.No.3, FGD.1). “I no longer go to work; I stay with the patient at home all the time” (FCG No.17, FGD.3).

**Table 3:** responses on the psycho-social and financial factors influencing role strain among family caregivers of adult patients with cancer

Variable		%				
Family caregiver related Psycho-social factors	F		F	%	F	%
I have become socially isolated from my family, friends and social events which strain me (social Isolation).	21.0	8.2	28	11.0	206	80.8
I experience sleep disturbance or lack of sleep due to caregiving which strain me (disturbed sleep cycle).	68.0	26.7	83	32.5	104	40.8
In the last two weeks, I have felt psychologically upset or experienced some stress which strains me (psychological distress).	37.0	14.5	21	8.2	197	77.3
I no longer get social support from the family/relatives and this strains me (lack of social support).	32.0	12.5	34	13.3	189	74.1
I experience anxiety and get fatigued which strains me (anxiety and fatigue).			36			80.0
**Family caregiver related financial factors**	15.0	5.9		14.1	204	
Paying for transportation during patient clinic attendance strains me (transportation costs).	89.0	34.9	50	19.6	116	45.5
Paying for accommodation during patient clinic attendance strains me (accommodation costs).	95.0	37.3	42	16.4	118	46.3
Buying chemotherapy drugs for the patient strains me (drug costs).	26.0	10.2	63	24.7	166	65.1
Paying for laboratory and radiological investigations ordered for the patient strains me (investigation costs).	14.0	5.5	69	27.1	172	67.4
Supporting the patient financially has made my financial status worse (general financial strain).	8.0	3.2	61	23.9	186	72.9

Notes: Each likert item was measured on a 5-point scale which were then computed as follows; disagree (strongly disagree + disagree) = did not perceive any strain; somehow agree slightly perceived the strain; agree (strongly agree + agree) = perceived the strain

The study also revealed that about two fifth (40.8%, n=104) of FCGs totally perceived a disturbed sleep cycle as a strain while 32.5% (n=83) slightly perceived the strain (those who somehow agreed) related to a disturbed sleep cycle. Indeed, during focus group discussions some respondents reported how caregiving had deprived them of sleep as supported by the following quote from one of the participants: “My patient is usually very weak for the first two weeks after receiving chemotherapy, I hardly sleep because I have to constantly check on my patient” (FCG No.10, FGD.2). The study also revealed that over three-quarters (77.3%, n=197) of FCGs were psychologically upset. More the study also revealed that about two fifth (40.8%, n=104) of FCGs totally perceived a disturbed sleep cycle as a strain while 32.5% (n=83) slightly perceived the strain (those who somehow agreed) related to a disturbed sleep cycle. Indeed, during focus group discussions some respondents reported how caregiving had deprived them of sleep as supported by the following quote from one of the participants: “My patient is usually very weak for the first two weeks after receiving chemotherapy, I hardly sleep because I have to constantly check on my patient” (FCG No.10, FGD.2).

The study also revealed that over three-quarters (77.3%, n=197) of FCGs were psychologically upset. Moreover, during focused group discussions majority of FCGs indicated that they experienced psychological distress related to caregiving as supported by the following quotes from some of the participants: “I get psychologically upset when I see how my daughter has been afflicted by cancer” (FCG No.6, FGD.1). “Right now I am stressed, my children are very young, I have called back home and been informed that they have not been prepared any meals” (FCG No.14, FGD.2). “My Patient feeds and vomits everything, this is stressing me and I do not know what type of food to give her” (FCG No.20, FGD.3).

Further, the study established that the majority (80.0%, n=204) of FCGs totally perceived strain related to anxiety and fatigue due to caregiving. In addition, during focus group discussions it was also revealed that caregiving predisposed FCGs to anxiety and fatigue as supported by the following quotes from some of the participants: “I am worried if my patient will make it…a number of things we do not understand, we are usually in the dark” (FCG.No.7, FGD.1). “…sometimes we have to go to town (Nairobi city) to buy the prescribed drugs that are not available at the hospital, we go searching from one chemist to another, this is tiresome, waste of time and expensive”(FCG.No.22, FGD.3).over, during focused group discussions majority of FCGs indicated that they experienced psychological distress related to caregiving as supported by the following quotes from some of the participants: “I get psychologically upset when I see how my daughter has been afflicted by cancer”(FCG No.6, FGD.1). “Right now I am stressed, my children are very young, I have called back home and been informed that they have not been prepared any meals” (FCG No.14, FGD.2). “My Patient feeds and vomits everything, this is stressing me and I do not know what type of food to give her” (FCG No.20, FGD.3).

Further, the study established that the majority (80.0%, n=204) of FCGs totally perceived strain related to anxiety and fatigue due to caregiving. In addition, during focus group discussions it was also revealed that caregiving predisposed FCGs to anxiety and fatigue as supported by the following quotes from some of the participants: “I am worried if my patient will make it,…a number of things we do not understand, we are usually in the dark” (FCG.No.7, FGD.1). “…sometimes we have to go to town (Nairobi city) to buy the prescribed drugs that are not available at the hospital, we go searching from one chemist to another, this is tiresome, waste of time and expensive” (FCG.No.22, FGD.3).

### Association between family caregiver-related psycho-social and financial factors with the role strain experienced by the family caregivers of adult cancer patients

Table 4 shows family caregiver-related psycho-social and financial factors associated with the role strain after multivariate ordinal logistic regression was performed. On psycho-social factors, the results established that family caregivers (FCGs) who did not perceive any strain (those who disagreed) related to social isolation were 80% less likely to experience severe role strain compared to those who totally perceived the strain (those who were in agreement) (OR=0.20, 95% CI 0.075-0.541, p=0.001). Likewise, FCGs who did not perceive any strain (those who disagreed) related to disturbed sleep cycle were 70% less likely to experience severe role strain compared to those who totally perceived the strain (those who agreed) (OR=0.30, 95% CI 0.141-0.641, p=0.002) while those who slightly perceived the strain (those who somehow agreed) were 46% less likely to experience severe role strain compared to those who totally perceived the strain (OR=0.54, 95% CI 0.299-0.982, p=0.044).

**Table 4:** results of ordinal logistic regression analysis on the association between family caregiver-related psycho-social/financial factors and family caregiver role strain among adult patients with cancer

Variable	Beta estimate	Sig.	Crude Odds Ratio	Beta estimate	Sig	Adjusted O.R	95% C.I	
							Lower	Upper
Bivariate ordinal logistic regression				Multivariate ordinal logistic regression				
**Family caregiver related psycho-social factors**								
I have become socially isolated from the family, friends and social events which strains me (social isolation)								
Disagree	-1.264	**0.004**	0.30	-1.606	**0.001**	0.20	0.075	0.541
Somehow agree	-0.801	**0.035**	0.45	0.541	0.222	1.72	0.722	4.084
Agree (Ref)								
I experience sleep disturbance related to caregiving which strains me (disturbed sleep cycle)								
Disagree	-1.633	**0.001**	0.20	-1.201	**0.002**	0.30	0.141	0.641
Somehow agree	-0.243	0.380	0.78	-0.612	**0.044**	0.54	0.299	0.982
Agree (Ref)								
In the last two weeks I have felt psychologically upset which strain me (psychologically stress)								
Disagree	-1.366	**0.001**	0.30	-0.057	0.905	0.94	0.375	2.380
Somehow agree	-0.467	0.277	0.63	0.543	0.308	1.72	0.606	4.894
Agree (Ref)								
I no longer get social support from family/friends and this strains me (lack of social support)								
Disagree	-1.226	**0.001**	0.30	-2.077	**0.001**	0.13	0.051	0.305
Somehow agree	-0.799	**0.023**	0.45	-1.868	**0.001**	0.15	0.061	0.391
Agree (Ref)								
Anxiety and fatigue related to caregiving strain on me												
Disagree	-1.120	**0.028**	0.33	0.222	0.717	1.30	0.375	4.166
Somehow agree	-0.654	**0.054**	0.52	0.108	0.803	1.10	0.477	2.606
Agree (Ref)								
**Family caregiver-related financial factors**												
Paying for transportation during patient clinic attendance strains me (transportation costs)								
Disagree	-0.845	**0.002**	0.43	-1.155	**0.017**	0.32	0.121	0.820
Somehow agree	-0.568	0.074	0.57	-0.621	0.194	0.54	0.211	1.372
Agree (Ref)								
Paying for accommodation during patient clinic attendance strains me (accommodation costs)								
Disagree	-0.500	**0.053**	0.61	0.368	0.397	1.45	0.618	3.380
Somehow agree	0.353	0.292	0.70	0.059	0.906	1.10	0.398	2.826
Agree (Ref)								
Buying chemotherapy drugs for the patient strains me (drug costs)								
Disagree	-0.170	0.665	0.84					
Somehow agree	0.148	0.592	1.20					
Agree (Ref)								
Paying for laboratory and radiological investigations ordered for the patient strains me (investigation costs)								
Disagree	0.067	0.897	1.20					
Somehow agree	0.016	0.953	1.02					
Agree (Ref)								
Supporting the patient financially has made my financial status worse (general financial strain)								
Disagree	0.265	0.695	1.30	0.819	0.259	2.30	0.547	9.412
Somehow agree	-0.638	**0.021**	0.53	-0.166	0.62	0.85	0.436	1.645
Agree (Ref)					4			

Notes: each Likert item was measured on a 5-point scale which was then computed as follows; disagree (strongly disagree + disagree) = did not perceive any strain; somehow agree slightly perceived the strain; agree (strongly agree + agree) = perceived the strain; only significant variables (p=>0.05) after bivariate logistic regression were considered for multivariate logistic regression; the dependent variable was the level of role strain (1=mild strain, 2= moderate strain, 3= severe strain (Ref).

Further, the study also revealed that FCGs who did not perceive any strain related to lack of social support from family and friends were 87% less likely to experience severe role strain than those who totally perceived the strain (OR=0.13, 95% CI 0.051-0.391, p=0.001), equally those who slightly perceived the strain (those who somehow agreed) were 85% less likely to experience severe role strain compared to those who totally perceived the strain (OR=0.15, 95% CI 0.061-0.391, p=0.001). This study did not establish any significant relationship between being psychologically upset and anxiety-fatigue related to caregiving with the role strain experienced by family caregivers of adult patients with cancer. Regarding financial factors associated with the family caregiver role strain, the results established that FCGs who did not perceive any strain (those who disagreed) related to transportation costs were 68% less likely to experience severe role strain compared to those who totally perceived the strain (those who agreed) (OR=0.32, 95% CI 0.121-0.816, p=0.017). This study did not establish any significant relationship between the accommodation costs and general financial strain with the role strain experienced by the family caregivers of adult patients with cancer.

## Discussion

This study has established that there are several family caregiver-related factors (socio-demographic and caregiving-related characteristics, psycho-social and financial) associated with the role strain experienced by FCGs of adult patients with cancer. Regarding socio-demographic and caregiving-related characteristics associated with the role strain, this study findings have revealed that being married (OR=0.49, 95% CI 0.252-0.960, p=0.038) was associated with less likelihood of experiencing severe strain while being unemployed (OR=3.29, 95% CI 1.833-5.894, p=0.001) was associated with more likelihood of experiencing severe strain. These study findings are consistent with another study [13] which revealed that being single contributed to high caregiving strain. Similarly, another study [14] in the U.S. established that being unemployed contributed to increased strain. However, there are other studies [13,15] that have established that being employed also contributed to high strain. These findings could likely be because family caregivers who are married have access to stronger and wider social support systems. Likewise, those who are employed are financially empowered and also have access to extra social support systems at the workplace.

This study also established that provision of care for less than 5 hours per day (OR 0.40, 95% CI 0.227-0.715, p=0.002) was associated with less likelihood of experiencing severe strain. This finding is in tandem with another study [16] in Malaysia which established that more caregiving hours is associated with high caregiving strain but another study [17] in the US established that there is no association. This finding could be attributed to role conflict with other responsibilities and lack of extra time for social and economic ventures which could cushion against role strain.

Regarding psycho-social factors associated with role strain, this study established that not perceiving social isolation as a strain (OR=0.20, 95% CI 0.075-0.541, p=0.001) was less associated with severe strain. This study finding is consistent with another study [18] in Denmark which established that limited social time with friends and family contributed to psychological distress. Similarly, another study [6] in Iran established that FCGs experienced disrupted social life and family-role conflict which greatly predisposed them to psychological distress. This study finding is likely because being socially isolated can lead to being disconnected from the social support systems which could cushion against the role strain. This study also revealed that not perceiving disturbed sleep cycle as a strain (OR=0.30, 95% CI 0.141-0.641, p=0.002) or slightly perceiving the strain (OR=0.54, 95% CI 0.299-0.982, p=0.044) was less associated with severe strain.

This finding is supported by another study [19] in Jordan which established that caregiving strain was associated with poor quality of sleep among FCGs of adult patients with cancer. This study finding is likely since the majority of patients with cancer receive chemotherapy and radiotherapy as an outpatient service and afterward return home where FGC continues monitoring the patient around the clock and thus has little time to rest. This current study also revealed that not perceiving lack of social support as a strain (OR=0.13, 95% CI 0.051-0.305, p=0.001) or slightly perceiving the strain (OR=0.15, 95% CI 0.061-0.391, p=0.001) was less associated with severe strain. This study finding is in line with another study [20] in Nigeria which established that social support (b=-0.137, p=0.001) significantly influenced caregiving strain. Similarly, another study [21] in India established that low perceived social support was associated with high caregiving strain. This study finding is likely since social support provides extra avenues for assistance and social support networks which could cushion against role strain.

On financial factors associated with role strain, this study established that lack of perception of transportation costs as a strain (OR=0.32, 95% CI 0.121-0.820, p=0.017) was less associated with severe strain. This finding is consistent with another study [6] in Iran which established that FCGs experienced financial strain related to transportation costs. Another study [22] in Ghana established that about 87% of FCGs experienced increased financial strain related to caregiving while 62% of FCGs reported that their finances had gotten worse. This study finding is likely because FCGs and their patients face increased transportation costs as they transverse wide geographical distances in search of cancer treatment services that are not available in their regions.

Study limitations: the study was conducted in only one care setting. The study also adopted a cross-sectional design and as such the study results cannot be generalized to the whole population. However, the study employed a large sample size. The study setting chosen is where the majority of patients with cancer and their family caregivers access comprehensive cancer treatment services from across the country. The study was based on respondents´ experiences which are subjective and could be affected by recall bias. However, the researcher employed the Modified Caregiver Strain Index (MCSI) tool [11] which is a validated tool that scores well even when screening for caregiver strain among long-term family caregivers.

## Conclusion

There are various family caregiver-related factors associated with role strain which include socio-demographic characteristics, psycho-social and financial factors. These can be a target for interventional research to mitigate caregiving strain among family caregivers of patients with cancer. We, therefore, recommend that healthcare practitioners proactively consider family caregivers for psychological counseling, social support groups, health education, and provision of literature materials on self-care and self-financial empowerment, spiritual support, and reference for financial support if available.

### What is known about this topic

- Family caregivers play critical roles in support of patients with cancer including some complex medical responsibilities that are usually performed by nurses and other medical personnel which impact patient care outcomes, yet there is no formalized way of integrating them in the unit of care;
- Family caregivers are at risk of caregiving strain, yet many cancers treatment centres do not support or offer them any skills training in caregiving;
- There is paucity of information regarding challenges faced by family caregivers of patients with cancer in Africa and interventional strategies in mitigating caregiving strain.

### What this study adds

- This study has revealed that there are various family caregiver related factors associated with role strain which include socio-demographic characteristics, psycho-social and financial factors which can be a target for interventional research to mitigate caregiving strain among family caregivers of patients with cancer;
- Nurses and other medical personnel who are always in direct patient care have a role of identifying family caregivers at risk of caregiver strain, offering them prompt psychological counselling, linking them with available supportive care, caregiving skills training, self-care and dissemination of educative literature materials.
